# Affordability of medicines in the European Union

**DOI:** 10.1371/journal.pone.0172753

**Published:** 2017-02-27

**Authors:** Tomasz Zaprutko, Dorota Kopciuch, Krzysztof Kus, Piotr Merks, Monika Nowicka, Izabela Augustyniak, Elżbieta Nowakowska

**Affiliations:** 1 Department of Pharmacoeconomics and Social Pharmacy, Poznan University of Medical Sciences, Poznań, Poland; 2 Department of Pharmaceutical Technology, Collegium Medicum in Bydgoszcz, Nicolaus Copernicus University in Toruń, Toruń, Poland; 3 Student Scientific Society, Department of Pharmacoeconomics and Social Pharmacy, Poznan University of Medical Sciences, Poznań, Poland; Jagiellonian University, POLAND

## Abstract

**Background:**

Medications and their prices are key issues for healthcare. Although access to medicines at affordable prices had been specified as a key objective of the European Health Policy, it seems that these goals have not been achieved. Therefore, we attempted an evaluation of affordability of selected medicines at full prices.

**Methods:**

The analysis concerned 2012 and was conducted between 2013 and 2015 in all the European Union (EU) countries divided into 3 groups depending on the date of their accession to the EU. Finally, we considered 9 originators used in the treatment of schizophrenia and multiple sclerosis. Information on drug prices were collected from pharmacies. Participation in the study was voluntary and anonymous in order to avoid accusations of advertising. To evaluate affordability, several factors were used (e.g. minimum earnings and Gini coefficient). Due to unavailability in some countries, the exact number of analyzed medicines varies.

**Results:**

Drug prices vary significantly between EU Member States. Almost eleven fold difference was observed between Germany (EUR 1451.17) and Croatia (EUR 132.77) in relation to Interferone beta-1a 22 μg. Generally, prices were the highest in Germany. The cheapest drugs were found in various countries but never in the poorest ones like Bulgaria or Romania. Discrepancies in wages were observed too (the smallest minimum wage was EUR 138.00 in Bulgaria and the highest EUR 1801.00 in Luxembourg). Full price of olanzapine 5mg, however, was higher in Bulgaria (EUR 64.53) than, for instance, in Belgium (EUR 37.26).

**Conclusions:**

Analyzed medications are still unaffordable for many citizens of the EU. Besides, access to medicines is also impaired e.g. due to parallel trade. Unaffordability of medications may lead to the patients’ non-compliance and therefore to increased direct and indirect costs of treatment. Common European solutions are needed to achieve a real affordability and accessibility of medications.

## Introduction

Medicines play a key role in healthcare and access to pharmacotherapy is a crucial human right [1, 2]. However, approximately one-third of the global population is unable to obtain the necessary medications [2, 3] and the price barrier is indicated as the main reason for this [1, 2]. Moreover, unaffordability of medicines is also related to the course and prevalence of serious and chronic diseases [3]; hence, the World Health Organization (WHO) recommends that healthcare decision-makers should incorporate suitable solutions to administer pricing policies and to ensure access to medicines [4].

Considering the importance of affordability and accessibility of medicines it is important to remember that medications are one of the most significant cost components of healthcare budgets. These costs are growing very fast due to population ageing in many Western countries and the increasing economic burden of new pharmaceutical technologies [5]. Apart from that, as a result of differences in healthcare systems or divergences in pharmaceutical companies strategies, prices of medicines could differ significantly even between neighboring countries e.g. within the European Union (EU) [5–7]. It may be the effect of different price levels or government policies related to tax rates, for instance [2]. Nevertheless, the European Commission has indicated equity and accessibility, sustainability and quality of care as the main health system objectives for the EU [8, 9]. Moreover, the US Department of Health and Human Services has emphasized close objectives in the strategic plan for a period between 2010 and 2015 [9].

Despite this and the fact that ensuring access to medicines at affordable prices is one of key objectives for the European Commission [10], it seems that these goals have not been achieved. Furthermore, due to discrepancies in prices of medicines among EU Member States, the barrier of unaffordable drugs is still a significant public health problem [2, 5].

Hence, we present a comparison of non-refunded drug prices and the evaluation of affordability of selected originator medicines used in the treatment of schizophrenia (SCH) and multiple sclerosis (MS). These disorders were selected because of their significant health and economic burden and because affordability and accessibility of medicines are crucial in attaining compliance and effective treatment of these chronic diseases [11, 12]. Both disorders are characterized by their economic burden related to direct, indirect and intangible costs [13, 14]. In the case of SCH a study carried out in 2002 in the US revealed that direct medical costs amounted to USD 22.7 billion versus 32.4 billion of indirect costs [15]. In addition to this, severity of SCH’s economic burden was confirmed by results from the UK where total cost of SCH was £ 6.7 billion [13]. For MS, however, the study conducted by Gustavsson et al. [16] indicated that total annual costs per subject ranged from EUR 29,423 in the Netherlands to 44,384 in France. Thus, these data confirm the importance of studies on cost-related aspects of the above-mentioned disorders. Besides, present study will allow an assessment of feasibility of EU goals considering the current situation on the drug markets which in some EU countries were severely affected by the financial crisis [2, 17].

## Materials and methods

The study presents the results of a retrospective drugs prices analysis conducted in all EU countries. Member States were divided into 3 groups (Table 1) related to the date of their accession to the EU. The first group includes 9 countries accessed before 1973 (Belgium, France, the Netherlands, Luxembourg, Germany, Italy, Denmark, Ireland, the United Kingdom). The second group includes 6 countries which joined between 1973 and 1995 (Greece, Spain, Portugal, Austria, Finland, Sweden). The last group includes 13 countries which joined between 1995 and 2013 (Cyprus, Czech Republic, Estonia, Lithuania, Latvia, Malta, Poland, Slovakia, Slovenia, Hungary, Bulgaria, Romania, and Croatia which joined the EU in 2013). The study was conducted between 2013 and 2015 and concerns 2012 price level.

**Table 1:** The comparison of GDP and GDP per capita as well as minimum and average wages in the EU.

**Countries—Group 1 (n = 9)**	**Average**	**SEM**	**SD**	**Median**	**Min**	**Max**
GDP [mln EUR] 2012	1014157.53	310583.85	931751.53	568650.81	40720.35	2529876.53
GDP per capita [EUR] 2012	33786.44	3957.45	11872.36	30733.00	23330	62698
minimum wage [EUR] 2012	1059.00	281.70	845.10	1398.00	1398^#^	1801
average wage [EUR] 2012	2106.33	117.07	351.20	2141.00	1597	2791
**Countries—Group 2 (n = 6)**						
GDP [mln EUR] 2012	362945.10	127680.10	312751.11	237556.78	156653.31	976315.82
GDP per capita [EUR] 2012	25146.83	4057.58	9939.00	25469.00	14409	38131
minimum wage [EUR] 2012	365.17	168.22	412.05	283.00	566^#^	877
average wage [EUR] 2012	1737.50	216.71	530.83	1881.00	907	2276
**Countries—Group 3 (n = 13)**						
GDP [mln EUR] 2012	76785.40*(0.0016)^ (0.0067)	26595.94	95893.03	37640.27	6454.79	361689.12
GDP per capita [EUR] 2012	11291.08*(<0.0001)^ (0.0051)	2274.95	8202.45	9573.00	1441	34218
minimum wage [EUR] 2012	379.62*(0.0110)	58.30	210.19	310.00	138	763
average wage [EUR] 2012	657.46*(<0.0001)^ (<0.0001)	90.80	327.39	659.00	190	1400
GDP [mln EUR] 2012	*p = 0*.*0023*					
GDP per capita [EURO] 2012	*p < 0*.*0001*					
minimum wage [EURO] 2012	*p = 0*.*0145*					
average wage [EURO] 2012	*p < 0*.*0001*					

* Statistically significant difference p < 0.05 vs. group 1

^ Statistically significant difference p < 0.05 vs. group 2

^#^ Data are partially available (in some countries included in this group the minimum wage was not regulated)

Preliminary sampling of 21 medicines has been conducted, allowing a final list of 9 originators in terms of brand names to be drawn up. 12 medicines were excluded as they had been virtually unavailable on the market in most of the countries and because there were some medicines representing other price categories originally included in the study, such as medicines known to be expensive and having a relatively low market share, and those with low prices and relatively high sales volumes. Originator drugs used in the treatment of SCH (Olanzapine 5mg and 10mg; Aripiprazole 15mg; Risperidone depot 25mg and 37.5mg) and MS (Interferone beta-1a 22μg, 30μg and 44μg; glatiramer acetate 20mg) were analyzed (Tables 2 and 3). The use of brand names is strongly limited to avoid any accusations of advertising, although these names are sometimes presented (Tables 2 and 3) to ensure clarity of the text.

**Table 2:** The comparison of P (EUR) and EA to medications used in the treatment of SCH.

**Zyprexa 5 mg–P (n = 26)**	**Average**	**SEM**	**SD**	**Median**	**Min**	**Max**
Group 1 (2012) (n = 9)	55.16	7.69	20.08	50.81	31.17	101.83
Group 2 (2012) (n = 6)	51.52	7.35	18.00	69.53	26.49	69.53
Group 3 (2012) (n = 11)	44.19	5.26	17.43	49.53	20.98	66.70
	*p = 0*.*4606*					
**Zyprexa 5 mg–EA (%) (n = 26)**	**Average**	**SEM**	**SD**	**Median**	**Min**	**Max**
Group 1 (2012) (n = 9)	2.72	0.41	1.23	2.85	1.49	4.66
Group 2 (2012) (n = 6)	3.12	0.48	1.18	3.06	1.94	5.24
Group 3 (2012) (n = 11)	10.08*(0.*0325)*	2.84	9.41	8.42	0.86	34.44
	*p = 0*.*0282*					
**Zyprexa 10 mg–P (n = 26)**	**Average**	**SEM**	**SD**	**Median**	**Min**	**Max**
Group 1 (2012) (n = 9)	105.77	14.32	42.97	99.74	61.43	182.74
Group 2 (2012) (n = 6)	104.29	18.70	45.81	97.625	53.00	177.85
Group 3 (2012) (n = 11)	77.63	10.66	35.34	74.29	27.09	134.29
	*p = 0*.*2509*					
**Zyprexa 10 mg–EA (%) (n = 26)**	**Average**	**SEM**	**SD**	**Median**	**Min**	**Max**
Group 1 (2012) (n = 9)	5.22	0.79	2.37	5.46	2.82	2.98
Group 2 (2012) (n = 6)	6.18	1.00	2.44	5.48	3.88	9.75
Group 3 (2012) (n = 11)	16.00*(0.*097)* *^(0*.*0487)*	3.29	140.91	15.97	1.67	36.30
	*p = 0*.*0067*					
**Abilify 15 mg–P (n = 25)**	**Average**	**SEM**	**SD**	**Median**	**Min**	**Max**
Group 1 (2012) (n = 9)	138.79	13.75	41.24	121.69	107.76	237.74
Group 2 (2012) (n = 6)	135.88	9.30	22.79	141.64	99.50	164.09
Group 3 (2012) (n = 10)	109.83	6.08	19.21	105.55	87.75	155.04
	*p = 0*.*0588*					
**Abilify 15 mg–EA (%) (n = 25)**	**Average**	**SEM**	**SD**	**Median**	**Min**	**Max**
Group 1 (2012) (n = 9)	6.71	0.68	2.05	5.90	4.61	10.87
Group 2 (2012) (n = 6)	8.26	0.78	1.91	7.52	6.52	10.97
Group 3 (2012) (n = 10)	24.31*(0.*0297)* *^(0*.*0146)*	5.54	17.52	19.45	3.48	66.52
	*p = 0*.*0059*					
**Rispolept Consta 25 mg–P (n = 20)**	**Average**	**SEM**	**SD**	**Median**	**Min**	**Max**
Group 1 (2012) (n = 7)	146.71	28.25	74.73	114.36	92.66	310.16
Group 2 (2012) (n = 5)	125.11	9.96	22.27	130.85	102.00	147.64
Group 3 (2012) (n = 8)	116.47	9.65	27.31	109.28	78.08	163.79
	*p = 0*.*4936*					
**Rispolept Consta 25 mg–EA (%) (n = 20)**	**Average**	**SEM**	**SD**	**Median**	**Min**	**Max**
Group 1 (2012) (n = 7)	6.84	1.26	3.32	5.26	4.92	14.18
Group 2 (2012) (n = 5)	8.12	1.14	2.54	6.98	5.75	11.18
Group 3 (2012) (n = 8)	26.87*(0.*0392)*	8.06	22.79	17.46	2.80	67.81
	*p = 0*.*0337*					
**Rispolept Consta 37.5 mg–P (n = 19)**	**Average**	**SEM**	**SD**	**Median**	**Min**	**Max**
Group 1 (2012) (n = 7)	206.11	44.41	117.51	147.37	129.44	462.78
Group 2 (2012) (n = 4)	173.76	16.72	33.44	183.01	127.16	201.86
Group 3 (2012) (n = 8)	151.69	10.06	28.46	146.17	115.63	212.55
	*p = 0*.*4017*					
**Rispolept Consta 37.5 mg–EA (%) (n = 19)**	**Average**	**SEM**	**SD**	**Median**	**Min**	**Max**
Group 1 (2012) (n = 7)	9.58	1.97	5.22	7.32	6.50	21.16
Group 2 (2012) (n = 4)	11.18	1.29	2.59	11.09	8.50	14.02
Group 3 (2012) (n = 8)	32.96*(0.*0321)*	8.91	25.19	25.01	4.14	85.25
	*p = 0*.*0348*					

Price (P) and economic affordability (EA). P (EUR) concerns 2012 price level.

* Statistically significant difference p < 0.05 vs. group 1

^ Statistically significant difference p < 0.05 vs. group 2

**Table 3:** The comparison of P (EUR) and EA to medications used in the treatment of MS.

**Rebif 22** μ**g–P (n = 21)**	**Average**	**SEM**	**SD**	**Median**	**Min**	**Max**
Group 1 (2012) (n = 9)	993.49	95.52	286.57	959.04	698.20	1451.17
Group 2 (2012) (n = 4)	982.28	69.52	139.04	992.57	802.51	1141.47
Group 3 (2012) (n = 8)	733.58	90.41	256.55	780.91	132.77	968.42
	*p = 0*.*1099*					
**Rebif 22** μ**g–EA (%) (n = 21)**	**Average**	**SEM**	**SD**	**Median**	**Min**	**Max**
Group 1 (2012) (n = 9)	48.33	5.38	16.14	45.08	29.91	75.04
Group 2 (2012) (n = 4)	57.77	10.39	20.78	49.68	43.62	88.48
Group 3 (2012) (n = 8)	131.37	39.86	112.74	112.30	13.42	375.15
	*p = 0*.*0685*					
**Rebif 44** μ**g–P (n = 26)**	**Average**	**SEM**	**SD**	**Median**	**Min**	**Max**
Group 1 (2012) (n = 9)	1204.27	136.60	409.80	959.04	718.53	1768.11
Group 2 (2012) (n = 6)	1108.96	77.43	189.67	1142.98	837.55	1324.68
Group 3 (2012) (n = 11)	961.86	36.55	121.21	942.03	799.35	1219.64
	*p = 0*.*1518*					
**Rebif 44** μ**g–EA (%) (n = 26)**	**Average**	**SEM**	**SD**	**Median**	**Min**	**Max**
Group 1 (2012) (n = 9)	58.70	7.76	23.29	48.21	34.54	100.90
Group 2 (2012) (n = 6)	68.54	9.48	23.21	56.16	50.76	102.93
Group 3 (2012) (n = 11)	198.68*(0.*0049)* *^(0*.*0287)*	38.83	128.77	164.79	37.14	450.41
	*p = 0*.*0025*					
**Avonex 30 μg–P (n = 21)**	**Average**	**SEM**	**SD**	**Median**	**Min**	**Max**
Group 1 (2012) (n = 9)	934.00	141.85	425.55	964.34	190.12	1664.03
Group 2 (2012) (n = 4)	966.26	112.23	224.46	943.40	738.00	1240.25
Group 3 (2012) (n = 8)	873.73	60.37	170.76	868.67	710.01	1247.77
	*p = 0*.*8736*					
**Avonex 30 μg–EA (%) (n = 21)**	**Average**	**SEM**	**SD**	**Median**	**Min**	**Max**
Group 1 (2012) (n = 9)	45.07	7.18	21.55	41.32	10.69	76.09
Group 2 (2012) (n = 4)	61.33	10.69	21.38	54.20	44.73	92.21
Group 3 (2012) (n = 8)	185.63*(0.*0115)*	51.41	145.40	148.30	25.66	487.18
	*p = 0*.*0146*					
**Copaxone 20 mg–P (n = 26)**	**Average**	**SEM**	**SD**	**Median**	**Min**	**Max**
Group 1 (2012) (n = 9)	1000.18	95.68	287.04	854.17	597.62	1489.76
Group 2 (2012) (n = 6)	922.43	83.93	205.58	900.39	685.21	1209.50
Group 3 (2012) (n = 11)	813.83	49.93	165.61	753.49	663.77	1185.78
	*p = 0*.*1952*					
**Copaxone 20 mg–EA (%) (n = 26)**	**Average**	**SEM**	**SD**	**Median**	**Min**	**Max**
Group 1 (2012) (n = 9)	48.47	5.31	15.93	41.07	33.59	75.53
Group 2 (2012) (n = 6)	56.05	6.35	15.54	52.73	41.53	85.17
Group 3 (2012) (n = 11)	171.78*(0.*0099)* *^(0*.*0445)*	38.26	126.88	130.66	24.54	480.38
	*p = 0*.*0060*					

Price (P) and economic affordability (EA). P (EUR) concerns 2012 price level.

* Statistically significant difference p < 0.05 vs. group 1

^ Statistically significant difference p < 0.05 vs. group 2

Although drugs in general are subject to reimbursement in all of the EU countries [7] comparison (Tables 2 and 3) took into account full prices of medicines as various price regulations in place in EU Member States could have a crucial impact both on the assumptions and results of the study. Moreover, cost of medicines may be subject to frequent changes in connection with reimbursement regimens.

Pharmacists from all EU countries were requested to provide relevant information on drug prices (in EUR or in local currencies which would be afterwards converted to the common European currency) in relation to the value paid by the patients. Considering the fact that the drugs’ dosage may differ among individuals, data about prices concerned one package of any medicine. If there were any differences in drug prices, the values obtained would be averaged. Potential study participants were selected from the list of pharmacies available on the websites concerning the study countries. Moreover, relevant addresses were gathered from tourist information and from the locals if necessary. Participation in the study was voluntary and anonymous in order to avoid accusations of advertising, which is strictly forbidden for pharmacies e.g. in Poland. Pharmacists were advised that information was collected for solely scientific purposes and that the study was anonymous thus, neither their names nor the name of the pharmacy would be recorded or disclosed. Therefore to ensure a common methodology and abide by the law, only information about the city where respondents worked was collected. Moreover, pharmacists might have been employed in more than one pharmacy, which would make it difficult for them to indicate their exact affiliation.

It was decided that information on drug prices would be collected from pharmacies located in centers of main cities in all EU countries as this is where the density of pharmacies is usually the highest. Pharmacies were chosen “accidentally” to avoid potential bias.

Data concerning selected drugs were generally obtained by direct contact; thus, our partners from other universities (Christian-Albrechts University in Kiel, Germany; Medical University of Plovdiv, Bulgaria; Poznan University of Life Sciences, Poland, for instance) as well as students were involved in the project and were requested to gather relevant information. In addition to this, data were also collected during our own stays in various EU countries.

Furthermore, in some cases, due to their daily responsibilities potential respondents would ask us to collect the data on the following day. If no reply would be given, the request would be reiterated twice. Each country was considered”completed” upon collection of the information from 3 different sources. In addition to that, both general and official national websites related to healthcare and medicines pricing were used to confirm the data obtained. Due to the lack of a proper feedback, Estonia was excluded from the study, and thus the final list of countries includes 27 EU Member States. Although Malta belongs to the group of included countries (we received a feedback from this Member State) no drug was actually analyzed there due to unavailability or the lack of data concerning the study medicines. Apart from the collected information on prices, data from Eurostat [18, 19], Organization for Economic Co-operation and Development (OECD) [20], and International Monetary Fund [21] concerning Gross Domestic Product (GDP), GDP per capita, average and minimum salaries (if applicable, as some countries such as Austria or Denmark have no minimum salary regulations) were used. To illustrate the potential disparities Gini coefficient (GC) was also used. In consideration of different currencies in several EU countries (e.g. Poland, Bulgaria, Czech Republic), money values were converted (using the 2013 exchange rate) from local currencies to the European currency (EUR) at the average EUR exchange rate published by the National Bank of Poland (NBP) on February 6, 2013, when the first response for our request had been obtained.

Presented herein is also economic affordability (EA) indicating the proportion of average remuneration needed to cover the full price of the drug. Although fluctuations in average incomes in EU countries are being observed annually, EA seems to be appropriate to evaluate affordability of medicines.

In some countries, prices of analyzed medicines were not available, hence the number of countries included in the analysis of individual drug varies. In the case of olanzapine, 26 countries were included and 25 countries in the case of aripiprazole. Risperidone depot 25mg was analyzed for 20 countries and 37.5mg–for 19 countries. In the case of Interferone beta-1a 22μg and 30μg, 21 countries were taken into consideration and for 44μg 26 countries were included–same as for glatiramer acetate.

This article does not contain any studies with human participants performed by any of the authors.

The data are shown as $\bar{x}$ ± SEM/SD, median with minimum and maximum values. Statistically significant results (p<0.05) were demonstrated for homogenous groups using 2-ways Anova test and post-hoc Tukey test.

## Results

Data for EU countries demonstrated a high variation both in terms of prices (P) and EA of medicines (statistical dependence is presented within Tables). For most of the chosen medications, P of the drug in the highest-priced country was significantly higher than in the location with the lowest P. The most significant discrepancy was observed in relation to Interferone beta-1a 22μg with the highest full price in Germany (EUR 1451.57) and the lowest price in Croatia (EUR 132.77). Otherwise, the smallest difference concerned Interferone beta-1a 44μg with the highest full price in Germany (1489.76 EUR) and the lowest in the Netherlands (EUR 718.53). What is interesting, all of the analyzed medicines turned out to be the most expensive in Germany. Considering the lowest price, however, olanzapine in both doses as well as aripiprazole were the cheapest in Slovenia. In general, risperidon depot had the lowest full price in Poland. Interferone beta-1a at the dose of 44μg as well as glatiramer acetate 20mg were the cheapest in the United Kingdom, at EUR 190.12 and EUR 597.62, respectively.

Of European countries (Table 1), in 2012 Bulgaria and Romania had the lowest level of monthly gross minimum wages at EUR 138 (average wage EUR 190.00) and EUR 162 (average wage EUR 297.00), respectively. On the other hand, countries ranking at the higher end, such as Luxembourg or Belgium, had the minimum monthly wage of EUR 1801 (average wage EUR 2334.00) and EUR 1444 (average wage EUR 2221.00) respectively (some EU Member States e.g. Austria or Finland have no national legislation setting a minimum wage). Nevertheless, in terms of prices of medicines such as the originator olanzapine 5mg, in Bulgaria the full price was EUR 64.53 but in Belgium it was EUR 37.26. Thus, this medication’s EA in Bulgaria was strongly impaired compared to a richer country. Apart from that, not once Bulgaria, Romania, Latvia, Lithuania, and other poorer countries turned out to be the countries with lowest prices of analyzed medicines. Some of the richest EU Member States, however, also happened to be the cheapest (the Netherlands or the UK, for instance).

Although earnings in new EU countries (Study group 3) have been increasing quite fast, disparities between the Member States are still significant (Table 1). The average level of minimum wages was almost 2.5 times higher in the group of the oldest EU Member States than in the group of new Member States. In terms of the level of average earnings, a threefold difference was observed between the first and the third group. Significant dissimilarities were also related to GDP and GDP per capita.

The collected data showed that EA of medicines was notably impaired between EU Member States reaching from three to fourfold difference between countries from the first and from the third group of EU. Nevertheless, the highest (4.11 times) and the smallest difference (2.71 times) in EA was observed in relation to Interferone beta-1a 30μg and 22μg, respectively. In terms of MS medications, the average EA related to full prices of medicines showed that patients from the third group of EU countries would not be able to buy a drug for their monthly earnings. In countries from the first and the second group, however, it would be a significant but probably still affordable expenditure, even at full price.

## Discussion

Despite the differences in healthcare organization and funding in various EU countries, health systems have the common primary objectives related to affordability and accessibility of medications [8, 9, 22]. Although these aims were pointed out already a few years ago [8, 10], it seems that these goals have still not been achieved.

While the access to several medicines has been increasing, affordability remains problematic, especially for poor populations [23]. It has been confirmed herein by presentation of huge discrepancies in P and EA (Tables 2 and 3) of the analyzed medicines. Full prices of medicines turned out not to be the lowest in the poorest (considering the level of earnings and GDP per capita) EU countries like Bulgaria, Romania or Latvia. On the contrary, P for some medicines was significantly higher or at the same level as in rich countries of the EU (Belgium or France, for instance). Interestingly, there are some governments and associations engaged in campaigns against “free riding” on high pharmaceutical prices in countries like the United States, for instance [24, 25]. This stems from the belief that poorer countries spend less per capita on pharmaceuticals than the richer ones. Thus they do not pay for research and development and are hence able to offer cheaper medications [24, 25]. Our findings corroborated with those of other authors [24] do not prove, however, “free riding” of the poorer countries at the expense of the affluent countries or of the pharmaceutical companies. Conversely, results of this study indicate the need for healthcare decision-makers to commit themselves to make drugs more affordable as in low-income countries the majority of citizens are unwilling to pay for effective pharmacotherapy due to the drug prices which are identical or even higher than in affluent countries [24, 26]. Furthermore, minimum or average wages were much lower in countries which joined the EU after 1995, reaching a thirteen fold difference between the citizens of the country with the lowest and the highest earnings. Our results showed that affordability of medicines could be significantly impaired for many EU citizens and these findings corroborate with previous comparisons of European prices [2, 5, 27].

In the study conducted by Vogler et al. [2], the authors showed a significant variation in medicine prices evaluated in New Zealand and 16 European countries reaching at least a twofold difference. Nevertheless, for a few medicines variation was significantly higher. These findings are convergent with the results hereof and with the report by Brekke et al. [28]. Moreover, these studies revealed that the United Kingdom or the Netherlands were countries with cheaper medications similarly to Spain, for instance. Germany or Belgium, however, as in our study, were on the other end of the scale [2, 28]. Interestingly, Brekke et al. [28] found that Norway (one of the richest countries in the world) had lower medicine prices than the Western European countries. It seems to confirm differences in medicine prices observed also in the present study. Notwithstanding, these divergences might be differently comprehended by implementing international dollars (I$) into the analysis [29]. This unit is a hypothetical currency aimed at explaining and comparing prices from one country to another. Costs in the local units are converted to I$ using Purchaisng Power Parity (PPP) exchange rate, which is the number of local currency units needed to get the same amount of goods a US $ would buy in the United States [30]. However, using PPPs rather than exchange rates may influence the perception of differences in drug prices leading to decrease (e.g. in Japan) and to increase (e.g. in Mexico) of price indexes, compared to the United States [31]. Nonetheless, considering affordability of medicines studied by Iyengar et al. [32] for most OECD countries at the nominal as well as PPP-adjusted prices analyzed drugs were not affordable, with Central and Eastern European countries being the most affected. In spite of the fact that I$ might be indicated as better unit for international comparisons, the currency exchange rates seem to be also acceptable and are used to conduct international comparisons of drug prices too [33].

Taking into consideration GC [34], however, the range of discrepancies observed could be contentious and differently interpreted. GC related to data from 2012 [34] was the lowest in Slovenia (country from a 3^rd^ group) at 23.7, followed by Sweden with 24.8, and Czech Republic (3^rd^ group) with 24.9. On the other hand, the highest GC was observed in Latvia at 35.7, followed by Portugal with 34.5, and Greece with 34.3. It is interesting that Luxembourg (the country with the highest minimum and almost highest average wages in EU) had GC 28.0 and Bulgaria (the country with the lowest minimum and average wages) 33.6. These data may result from the global financial crisis, which strongly hit EU countries especially from the Mediterranean area [2, 17]. Nonetheless, the differences in GDP and GDP per capita seem to confirm divergences in the economic status of EU Member States, hence prices of medicines should be adjusted to local economy and should guarantee essential affordability [1, 23], especially in countries where this key factor is impaired.

A solution which could compensate differences in P and EA between EU countries is parallel trade. Although this well known idea may lead to the increase of affordable access to medicines, it could have an opposite impact as well [27, 35, 36]. It is related, among other things, to the market controlled by pharmaceutical companies which limit access to crucial medicines [5] and this situation is being observed e.g. in Poland nowadays. On the other hand, divergences in medicine prices may result in the development of generic substitution, which is generally associated with relevant monetary savings [31, 37, 38]. Generic substitution, however, is not a perfect solution and according to the Himmel et al. [39] and Hassali et al. [40] there are a lot of patients who reported more severe side effects of substituted pharmacotherapy and expressed concerns related to using generic brands instead of originator drugs. Nevertheless, extensive use of generic brands of medicines which contain the same and bioequivalent substance as the originators could significantly improve affordability of medicines [38, 41]. Furthermore, countries which had generic competition as well as several options to increase generic uptake, succeeded in bringing generic medicine prices down [2]. Therefore, competition between generics and originators affects availability and improves affordability of medicines [2].

The range of discrepancies especially in terms of affordability of medicines may be related to factors significantly affecting the differences in pharmaceutical prices. The level of national income per capita and national regulatory approaches are identified as key issues [5]. Another important factor is that VAT rates on medications vary between the EU Member States, being as high as 25% in Denmark, 20% in Bulgaria, 19% in Germany, or as low as 10% in Italy, 8% in Poland, and 5% in Lithuania [5, 42]. In Sweden, meanwhile, pharmaceutical products are exempt from VAT [5, 42]. In addition to this, profit margins for medicines in some countries are statutory (mostly on refundable medicines, although 5 EU countries apply price control to all medications), which may intensify the differences in medications’ prices between EU Member States [7].

Considering the affordability of medicines, it is important to point out that all EU countries have reimbursement lists [7], thus the affordability of many medications seems to be significantly improved. The Member States, however, have different reimbursement policies in place in terms of co-payment which has increased in the last few years [2, 5, 17] and, as a response to the global financial crisis, has turned out to be an affordability barrier as well [2, 5]. Reimbursement lists can also be updated at different intervals (e.g. every two months in Poland and every month in Spain), and thus pharmaceutical companies may withdraw their products from reimbursement lists to avoid being subject to price control (in Poland, for instance, original olanzapine in oral solid was reimbursed but since September, 2014 is no longer subject to reimbursement) and to restrain parallel trade. Importantly, there are patients within the EU who are obliged to cover full price of medicine due to the lack of health insurance or because of the off-label indications. Thus, it seems important to analyze prices of medicines without reimbursement. Moreover, from our point of view, a study related to full prices of medicines provides interesting information also for third-party payers, HTA agencies and people responsible both for local health policies and for the common European health policy.

Our findings corroborate with recommendations of WHO experts [4] who indicate that policymakers should manage medicine prices to provide accessible and affordable medicines to the community and individuals likewise. Despite this, availability and affordability of medicines are unsatisfactory [34, 43]. Although the EU Member States have the right to regulate prices of medicines individually [2, 7], there is a need to put common European strategies in place to guarantee affordable prices of medicines satisfactory for the payers, pharmaceutical companies, and the patients in particular. Considering the economic factors and statistics, it is questionable that the same drug should be more expensive or have a similar price in low- or middle-income EU countries as in the richest Member States. In this context, EU decision makers could encourage pharmaceutical companies and support local politicians to provide significant price reductions in a number of European markets. It should ensure delivery of the main objectives of EU public health policy related to free access to medicines at an affordable level [10].

Our study has some limitations, though. It is based on few medicines so our findings can only provide an indication concerning affordability. It could be interesting to analyze more medications and generics, too. Still, presentation of such results within a single study would be impossible. Therefore, disorders were selected which require compliance and are frequently associated with productivity loss which may additionally increase the lack of affordability. Moreover, the price survey is based on full prices of a single package of any medicine and the authors are aware of the fact that it would be reasonable to take reimbursement regulations into account. Nevertheless, analysis of all EU Member States’ reimbursement policies could make the study incomprehensible. Besides, patients are sometimes obligated to pay full prices for medicines also due to the lack of reimbursement for a drug in their EU country. Furthermore, medicines could be withdrawn from the reimbursement list and the level of co-payment might change as much as several times a year as a result of different intervals related to the update of reimbursement lists. It could also be interesting to consider the monthly cost of therapy. Nevertheless, due to the differences in drugs’ dosage and frequently customized pharmacotherapy, prices one of a single package were analyzed. Besides, although the study concerns several coefficients and economic factors, it could be interesting to take into consideration PPP and data related to countries from European Free Trade Association (EFTA) as well. Moreover, the use of I$ may improve the study. Despite that I$ is a hypothetical currency, the unit is used to compare the values of different currencies. Nonetheless, drug prices might be compared using different indices too. In the effect it can help nations to establish a suitable pricing system as well as provide more affordable medicines. Furthermore, the present study is related to the EU market, thus we decided to use the common European currency, which is adopted in 19 out of 28 EU countries. Moreover, though numerous economic factors are frequently presented in USD we chose to use the European currency (EUR) to ensure clarity of the text primarily for European readers who are not specialists in the field. Nevertheless, researchers, pharmaceutical industry representatives, and stakeholders should likewise be interested in thus presented results.

To our knowledge there is still an insufficient number of similar analyses, which makes this study even more relevant. Nevertheless, further research is necessary to extend the study onto a larger sample of medicines.

## Conclusions

In spite of the fact that the European Commission identified affordability and accessibility of medicines as key objectives of the public health policy, crucial medications are frequently still unaffordable for many EU citizens, especially at full prices. This suggests that greater affordability would require a review of the pricing policies and regulatory structures also in relation to the EU as a whole. Prices of medications should be adjusted to local economies. Differences in prices may lead to impaired access to medicines due to parallel trade and pharmaceutical companies’ policies which may restrain access to pivotal medications. Unaffordability of medications may contribute to non-compliance and, thus, may lead to an increase in both direct and indirect costs of treatment. There is a need for all-European solutions which will guarantee the affordability of medicines and profitability for companies and support national pricing negotiations. It should contribute to bringing prices of medicines to levels adequate to domestic economic factors and lead to achievement of the main goals of the European public health policy.

## Supporting information

S1 Table List of drugstores location. (DOCX)

S2 Table Affordability data. (XLSX)
