# Supplementary material for: Affordability of medicines in the European Union
Source: PLoS One. 2017 Feb 27;12(2):e0172753. doi: 10.1371/journal.pone.0172753 (PMC5328386; doi:10.1371/journal.pone.0172753)
Supplement: S1 Table — (DOCX) [file pone.0172753.s001.docx]

| Country | City |
| --- | --- |
| I group |  |
| Belgium | Brussels (3x) |
| France | Paris (3x) |
| The Netherlands | Amsterdam (2x), Rotterdam (1x) |
| Luxembourg | Luxembourg (3x) |
| Germany | Kiel (3x) |
| Italy | Rome (3x) |
| Denmark | Copenhagen (3x) |
| Ireland | Dublin (3x) |
| UK | London (3x) |
| II group |  |
| Greece | Athens (3x) |
| Spain | Barcelona (2x), Madrid (1x) |
| Portugal | Porto (3x), |
| Austria | Wien (3x) |
| Finland | Helsinki (2x), Turku (1x) |
| Sweden | Stockholm (3x), |
| III group |  |
| Cyprus | Nicosia (1x), Limassol (2x) |
| Czech Republic | Prague (3x) |
| Estonia | Exluded from the study – Lack of feedback |
| Lithuania | Vilnus (3x) |
| Latvia | Riga (3x) |
| Malta | Valetta (3x) |
| Poland | Poznan (3x) |
| Slovakia | Bratislava (3x) |
| Slovenia | Ljublana (3x) |
| Hungary | Budapest (3x) |
| Romania | Bucharest (3x) |
| Bulgaria | Plovdiv (3x) |
| Croatia | Split (2x), Zagreb (1x) |
